# Diversity of Bacteria Associated with Guts and Gonads in Three Spider Species and Potential Transmission Pathways of Microbes within the Same Spider Host

**DOI:** 10.3390/insects14100792

**Published:** 2023-09-29

**Authors:** Yue Liu, Jia Liu, Xiaopan Zhang, Yueli Yun

**Affiliations:** 1 State Key Laboratory of Biocatalysis and Enzyme Engineering, Hubei Collaborative Innovation Center for Green Transformation of Bio-Resources, School of Life Sciences, Hubei University, Wuhan 430062, China; 2 Centre for Behavioral Ecology & Evolution, School of Life Sciences, Hubei University, Wuhan 430062, China

**Keywords:** microbial communities, spider gut, spider gonad, 16S ribosomal RNA

## Abstract

**Simple Summary:**

Despite extensive research into the microbial communities of insects, studies on spiders, an important arthropod group, have been relatively limited in the field of microbiology. This study focuses on the analysis of gut microbiota and gonad microbiota from three different spider species. Using high-throughput sequencing, we have identified variations in microbial composition among different spider species, particularly notable differences between female and male spiders. Sequencing results also suggest similarities between the gut and ovarian microbiota of spiders, implying potential pathways for microbial transmission within female spiders between the gut and gonad. This research provides a theoretical foundation for exploring the relationship between microbes and spider hosts, as well as the role of microbes in spider physiology, ecology, and behavior.

**Abstract:**

Microbial symbiosis plays a crucial role in the ecological and evolutionary processes of animals. It is well known that spiders, with their unique and diverse predatory adaptations, assume an indispensable role in maintaining ecological balance and the food chain. However, our current understanding of spider microbiomes remains relatively limited. The gut microbiota and gonad microbiota of spiders can both potentially influence their physiology, ecology, and behavior, including aspects such as digestion, immunity, reproductive health, and reproductive behavior. In the current study, based on high-throughput sequencing of the 16S rRNA V3 and V4 regions, we detected the gut and gonad microbiota communities of three spider species captured from the same habitat, namely, *Eriovixia cavaleriei*, *Larinioides cornutus*, and *Pardosa pseudoannulata*. In these three species, we observed that, at the phylum level classification, the gut and gonad of *E. cavaleriei* are primarily composed of Proteobacteria, while those of *L. cornutus* and *P. pseudoannulata* are primarily composed of Firmicutes. At the genus level of classification, we identified 372 and 360 genera from the gut and gonad bacterial communities. It is noteworthy that the gut and gonad bacterial flora of *E. cavaleriei* and *L. cornutus* were dominated by *Wolbachia* and *Spiroplasma*. Results show that there were no differences in microbial communities between females and males of the same spider species. Furthermore, there is similarity between the gut and ovary microbial communities of female spiders, implying a potential avenue for microbial transmission between the gut and gonad within female spiders. By comprehensively studying these two microbial communities, we can establish the theoretical foundation for exploring the relationship between gut and gonad microbiota and their host, as well as the mechanisms through which microbes exert their effects.

## 1. Introduction

Arthropods contain a large number of microorganisms. The gut microbiota, through long-term coevolution with their hosts, has developed highly diverse population structures and intricate biological functions. These microbiota play a significant role in various aspects of the host, including development, reproduction, stress resistance, and behavior [1,2,3,4,5,6]. Among them, insects, as a crucial group within the Arthropoda, have had their gut microbiota diversity extensively investigated in various studies, including termites [7,8,9], aphids [10,11], butterflies [12,13], and honey bees [14,15,16], among others. As another significant subgroup within the arthropods, spiders have relatively limited research in the field of gut microbiota [17,18,19,20].

Spiders are important natural enemies of agricultural pests and can be used for biological control in crops and orchards [21,22,23]. Currently, most studies on microorganisms within spiders have focused on endosymbionts, which include *Wolbachia*, *Cardinium*, *Spiroplasma*, and *Rickettsia*, among others [24,25,26,27,28,29,30,31]. Some endosymbionts have been found to affect the reproductive system of spiders by regulating the sex ratio and so on, thus having an important impact on their reproduction [32,33,34,35,36]. In addition, some microorganisms might form symbiotic relationships within arthropod reproductive systems, positively influencing the reproductive health and reproductive success of the host [37,38]. In summary, microorganisms residing within arthropod reproductive tissues or transmitted through arthropod reproductive systems have profound impacts on the host’s reproduction, health, and evolution [39].

To investigate the diversity of gut and gonad microbiota in spiders and explore variations in gut microbiota among spiders with different predatory strategies, we selected three spider species (*Eriovixia cavaleriei*, *Larinioides cornutus*, and *Pardosa pseudoannulata*) from the same habitat as the focal species for this study. Among them, *Eriovixia cavaleriei* and *Larinioides cornutus* belong to the family Araneidae and are orb-weaver spiders, while *Pardosa pseudoannulata* belongs to the family Lycosidae and is a wandering hunting spider. These two spider species from different families exhibit distinct predatory strategies. In the study by Lihua Zhang [40], an analysis of endosymbionts and bacterial communities within various spider species has been conducted, revealing differences in the abundance of endosymbionts and other bacteria among them. Therefore, it is crucial to screen the microbial communities present in the gut and gonads of different spider species, as well as determine their effects on the host. Based on this, we explored the following questions for the spider microbial community: (I) What is the composition of gut and gonad microbial communities in the three spider species? Are there differences in the gut or gonad microbial communities among the three spider species? (II) Are there differences in the gut or gonad microbial communities between female and male spiders of the same host species? (III) Is there a potential link between gut and gonad microflora? To answer these questions, we employed high-throughput sequencing techniques to investigate the diversity and composition of gut and gonad microbial communities in the three spider species. We also compared the differences in microbiota not only between the female and male gut but also between the gut and gonads of the same spider individuals. The results of this study will contribute to a deeper understanding of the gut and gonad microbiota of spiders, as well as provide important clues about the possibility of microbiota transmission within the same host individual.

## 2. Materials and Methods

### 2.1. Samples

*E*. *cavaleriei* (Araneidae), *L*. *cornutus* (Araneidae), and *P*. *pseudoannulata* (Lycosidae) are common and easily collected spider species found near Sand Lake, Wuhan, China. Adult spiders were collected from the field in August 2017. All living samples were transported to the laboratory and starved for at least 7 d to avoid the influence of prey microorganisms. The gut and gonads of each spider species were then removed and dissected. All samples were visually identified under a microscope. The spiders used in this study were all non-endangered and non-protected species.

### 2.2. Dissection and DNA Extraction

**Figure 1:**
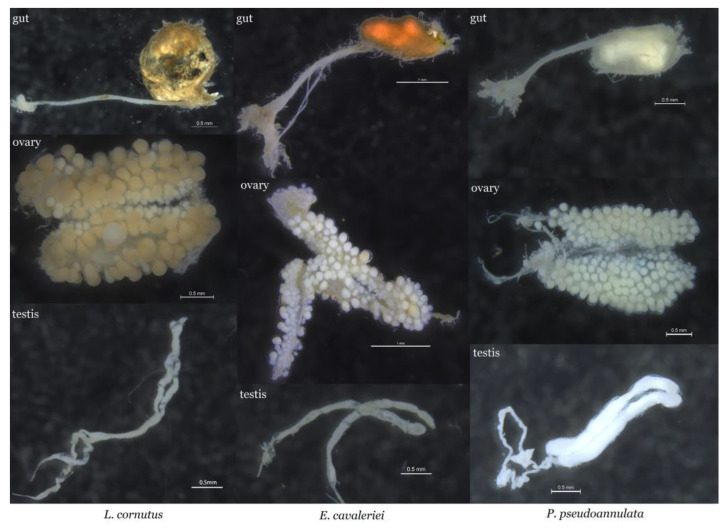
Gut and ovary anatomy in three spider species.

We selected 15 females and 15 males from each spider species. Each spider was rinsed with 1% sodium hypochlorite for 1 min, 75% ethanol for 1 min, and sterile ultrapure water for 3 min. Samples were placed in clean petri dishes (90 mm in diameter) with sterile fine-tip forceps under a stereomicroscope, and the gut and gonads were aseptically dissected from the abdomens in sterile phosphate-buffered saline (PBS). The gut and ovary anatomy of the three spider species is shown in Figure 1. Gut samples from five female individuals from the same spider species were pooled into one 1.5-mL microcentrifuge tube as a female gut sample, and ovaries from the same female spiders were pooled into one 1.5-mL microcentrifuge tube as an ovary sample. Three replicates were established for the female gut and ovary samples, respectively. Gut samples from five male individuals from the same spider species were pooled into one 1.5-mL microcentrifuge tube as a male gut sample, and testes from the same male spiders were pooled into one 1.5-mL microcentrifuge tube as a testis sample. Three replicates were established for the male gut and testis samples, respectively. Information on all samples is shown in Table 1. The gut and gonad samples were stored at −80 °C for DNA extraction. The DNA of each sample was extracted using a DNeasy Blood and Tissue Kit (Qiagen, Dusseldorf, Germany) following the manufacturer’s standard protocols. DNA sample quality was checked by electrophoresis on a 1.5% agarose gel and then stored at −20 °C for polymerase chain reaction (PCR) amplification of bacteria.

### 2.3. PCR Amplification and Sequencing

The bacterial diversity of the samples was assessed by amplifying the V3 and V4 regions of the 16S rRNA gene using primers (338F: 5′-ACTCCTACGGGAGGCAGCA-3′ and 806R: 5′-GGACTACHVGGGTWTCTAAT-3′) [41]. The PCR mixture contained 15 μL of Phusion High-Fidelity PCR Master Mix (New England Biolabs, Ipswich, UK), 1 μL of each forward and reverse primer (10 μM), 1 μL of template DNA (100 ng/μL), and deionized ultrapure water adjusted to 30 μL. The PCR thermal cycling program included an initial denaturing step at 94 °C for 5 min, followed by 35 amplification cycles at 94 °C for 30 s, 55 °C for 45 s, 72 °C for 45 s, and a final extension step at 72 °C for 7 min on an Applied Biosystems thermal cycler (MyCler BIO-RAD, Hercules, CA, USA). The PCR products were verified by running the samples on a 1.5% agarose gel. The amplicon products were subjected to unidirectional pyrosequencing on an Illumina HiSeq 2500 high-throughput sequencing platform under contract with BioMarKer Technologies, Beijing, China.

### 2.4. Bioinformatics and Statistical Analysis

Paired-end reads were merged into single sequences with the removal of barcodes and primers using FLASH (v1.2.7) [42]. Based on Trimmomatic software (v0.33), quality filtering of the raw tags was performed to obtain high-quality, clean tags. Final effective tags were obtained using UCHIME (v4.2) to detect and remove chimeric sequences (Mothur v1.31.2) [43]. Sequence analysis was performed using QIIME2 software to assign microbial diversity via the construction of an operational taxonomic unit (OTU) table. The OTUs were defined at a 97% sequence similarity threshold with the average neighbor clustering algorithm. Simultaneously, the most abundant sequence of each OTU cluster was selected and screened for further annotation under the UCLUST algorithm [44]. The OTU taxonomy was assigned to the RDP Classifier (v2.2) using the Silva databases [45]. Raw reads were submitted to the NCBI Sequence Read Archive (SRA) database (Accession numbers: PRJNA550176).

Alpha diversity indices (i.e., Ace, Chao 1, Shannon, and Simpson), which indicate species richness and community diversity of samples, were calculated in QIIME (v1.8.0). Beta diversity was determined with binary_jaccard. The differences in microbial communities were resolved using R software (v2.15.3) [46]. All statistical analyses were conducted using the R statistical computing environment (R v3.3.1). To determine the possible differences between groups, we conducted analysis of variance (ANOVA) and performed Bonferroni post hoc tests for multiple comparisons.

## 3. Results

### 3.1. Comparative Morphological Analysis of the Gut and Gonads in Three Spider Species

The midgut of *E. cavaleriei* is slender, with a posterior enlargement forming an oval-shaped dung sac. The intestinal wall and the area around the dung sac exhibit numerous small branches. Its ovaries are closely paired, containing white egg grains. The ovaries are slender in shape, and the testes are located at the same position within the spider’s body. In *E. cavaleriei*, the testes are paired and shorter in length compared to the intestinal tract (Figure 1). In contrast, *L. cornutus* exhibits a more elongated midgut with a posteriorly enlarged dung sac that takes on a larger oval shape. Numerous small branches surround the dung sac, and the intestinal wall is thin, allowing the contents of the dung sac to be clearly visible. Its ovaries are closely paired, and the testes are slender, connected to even more elongated and convoluted sperm ducts (Figure 1). As for *P. pseudoannulata*, its midgut is relatively short, and the intestinal tract and dung sac are surrounded by numerous small branches. The ovaries of *P. pseudoannulata* are paired, and the egg grains are more rounded. Its testes are larger than the intestinal tract and are connected to a pair of curved, elongated sperm ducts (Figure 1).

### 3.2. The Diversity Analysis of the Bacterial Community

A total of 2,464,420 raw reads from the gut and gonads of the three spider species were characterized using Illumina high-throughput sequencing. After quality filtering, 1,464,521 high-quality clean tags were obtained and grouped into 1930 gut bacteria OTUs and 1985 gonad bacteria OTUs based on a 97% similarity distance level. We used Shannon, Simpson, Chao1, and ACE indices to evaluate alpha diversity across the different bacterial communities in the three spider species (Table 2). Statistical analyses of species richness and community diversity indices indicate that there are no significant differences in bacterial community diversity and species richness between the male and female gut microbiota of *E. cavaleriei* (Shannon, *p* = 0.9540; Chao1, *p* = 0.7548), *L. cornutus* (Shannon, *p* = 0.8927; Chao1, *p* = 0.8047), and *P. pseudoannulata* (Shannon, *p* = 0.2302; Chao1, *p* = 0.2276). Among the three spider species, the gut bacterial community diversity (Shannon) is higher in *L. cornutus* and *P. pseudoannulata* compared to *E. cavaleriei*. Bacterial community diversity was significantly higher in the ovary than in the testis of *E. cavaleriei* (Shannon, *p* < 0.05), and there were no significant differences in bacterial community diversity and species richness between the ovary and testis of *L. cornutus* (Shannon, *p* = 0.3269; Chao1, *p* = 0.8782) and *P. pseudoannulata* (Shannon, *p* = 0.4432; Chao1, *p* = 0.9281). Among the three spider species, the ovary and testis bacterial community diversity (Shannon) is higher in *L. cornutus* and *P. pseudoannulata* compared to *E. cavaleriei*. In *E. cavaleriei*, there was no significant difference in the species richness of bacteria within the ovaries or testes when compared to *L. cornutus* or *P. pseudoannulata* (*p* > 0.05). However, the species richness of bacteria within the testes of *P. pseudoannulata* was significantly higher than that within the testes of *L. cornutus*, as indicated by Chao1 analysis (Chao1, *p* < 0.05). Overall, the gut bacterial communities of these three spider species exhibit greater diversity than the bacterial communities within their gonads.

**Figure 2:**
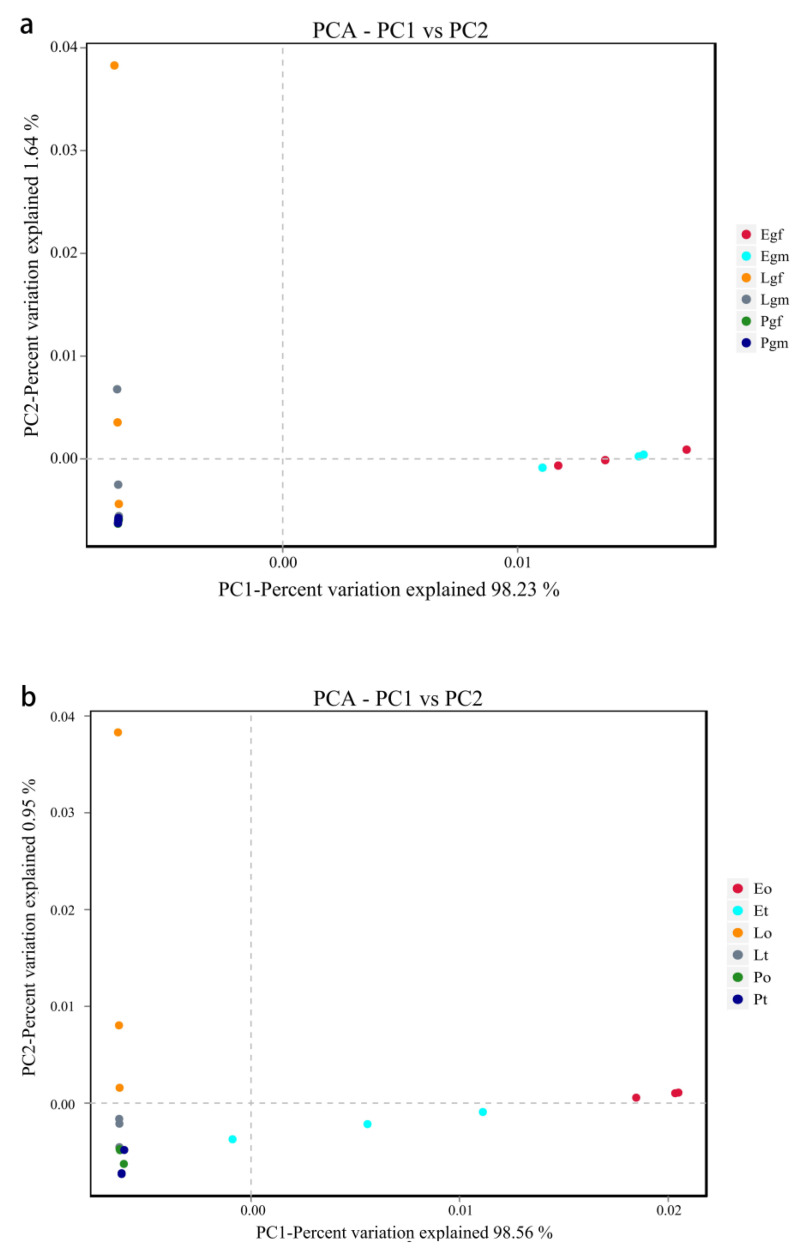
PCA of bacterial communities in the gut (**a**) and gonads (**b**) of three spider species. Note: The specific names corresponding to the legend sample ID are presented in Table 1.

Based on principal component analysis (PCA), the first two components (Figure 2a), PC1 and PC2, accounted for 99.87% of the differences (PC1 = 98.23%, PC2 = 1.64%) among gut bacteria in the three spider species. As can be seen in Figure 2, female and male gut samples of *E. cavaleriei* clustered together, and both male and female gut samples of *L. cornutus* and *P. pseudoannulata* clustered together (except for one dot). Among the three spider species, the gut samples of *E. cavaleriei* are significantly separated from the gut samples of *L. cornutus* and *P. pseudoannulata*. The first two components (Figure 2b), PC1 and PC2, also accounted for 99.51% (PC1 = 98.56%, PC2 = 0.95%) of the differences between the ovary and testis bacteria in the three spider species. We found that ovary and testis samples from *E. cavaleriei* were separated from each other, ovary and testis samples from *L. cornutus* were clustered together (except for one point), and gonad samples from *P. pseudoannulata* were clustered together. Similar to the gut samples, among the three spider species, the gonad samples of *E. cavaleriei* are significantly separated from the gonad samples of *L. cornutus* and *P. pseudoannulata*. In summary, the β-diversity results from PCA indicate that the gut bacterial community structures of male and female *E. cavaleriei* are quite similar, while there are certain differences in the bacterial community structures between the ovary and testis. However, both *L. cornutus* and *P. pseudoannulata* spiders did not exhibit significant differences in either gut bacterial community structure or gonad bacterial community structure.

### 3.3. Composition and Differential Analysis of Gut and Gonad Bacterial Communities

**Figure 3:**
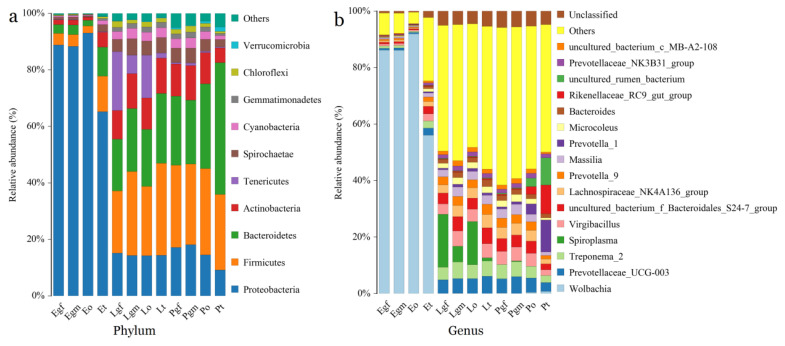
Relative abundances of bacteria in the gut and gonads of three spider species. (**a**) At phylum level; (**b**) At genus level. Note: The specific names corresponding to the sample ID on the *x*-axis are provided in Table 1.

The phylum Proteobacteria accounted for the majority of reads from the gut and gonads in *E. cavaleriei*, and Proteobacteria abundance in the ovary of *E. cavaleriei* reached 93.08% (Figure 3a). Firmicutes was the dominant phylum in the gut and gonad samples of *L. cornutus* and *P. pseudoannulata*, followed by Bacteroidetes and Proteobacteria (Figure 3a). At the genus level, 372 and 360 genera were detected with 97% similarity in all gut and gonad samples, respectively. *Wolbachia* was the dominant genus in the gut and gonads of *E. cavaleriei* (Figure 3b). In contrast, the abundances of endosymbionts *Wolbachia* and *Spiroplasma* in the gut and gonads of *P. pseudoannulata* were ≤1%, whereas *Spiroplasma* dominates in the female gut and ovarian samples of *L. cornutus*, as depicted in Figure 3b.

As shown in Table 3 and Table 4, in the gut of both female and male *E. cavaleriei*, as well as in the ovaries and testes, the relative abundance of the top 30 bacterial taxa shows no significant differences (*p* > 0.05). In *L. cornutus*, there were no significant differences (*p* > 0.05) in the relative abundance of the top 30 bacterial taxa between the ovaries and testes. With the exception of *Lachnospiraceae_NK4A136_group*, *uncultured_bacterium_f_Ruminococcaceae*, *Bacteroidales_S24-7_group*, and *Treponema_2*, the remaining bacterial taxa in the gut of both female and male *L. cornutus* also show no significant differences (*p* > 0.05). In *P. pseudoannulata*, there were no significant differences (*p* > 0.05) in the relative abundance of the top 30 bacterial genera between the ovaries and testes, as well as between the female and male gut. Overall, there were no differences in the gut and gonad bacterial community structures between the males and females of the three spider species.

Among the three spider species, at the phylum level, the relative abundance of Proteobacteria in the gut and gonads of *E. cavaleriei* was significantly higher than that in *L. cornutus* and *P. pseudoannulata* (*p* < 0.05). In contrast, the relative abundance of certain other phyla, such as Firmicutes, Bacteroidetes, and Actinobacteria, in *E. cavaleriei* was lower than in *L. cornutus* and *P. pseudoannulata* (Table 3 and Table 4).

### 3.4. Differences in Relative Abundance of Bacteria between Spider Gut and Gonad in Males and Females

As shown in Table 5, based on the top 30 abundant bacteria in each spider species, we analyzed the differences between the female gut and ovaries, as well as the male gut and testes, in the same species of spider. We found that the relative abundances of two genera in the top 30 bacteria show significant differences between the female gut and ovary in *E. cavaleriei*, whereas the relative abundances of 21 genera in the top 30 bacteria show significant differences between the male gut and testis. In *L. cornutus* and *P. pseudoannulata*, there were no significant differences in the relative abundances of bacteria between the female gut and ovary in the top 30 bacteria genera, whereas the relative abundances of three genera in *L. cornutus* and ten genera in *P. pseudoannulata* demonstrate significant differences between the male gut and testis. These results suggest that the bacterial communities in the female gut were similar to those in the ovary in the same spider species, whereas the communities in the male gut demonstrate significant differences as compared to those in the testes in the same spider species.

## 4. Discussion

Here, based on high-throughput sequencing, we identified 372 and 360 genera in the gut and gonad bacterial communities, respectively, of the three spider species. The research findings indicate that among the gut and gonad microbiomes of these three spider species, bacteria from the phyla Proteobacteria, Firmicutes, and Bacteroidetes are the most significant constituents. This finding is not only well-supported within the field of spiders [19,47,48], but also has similar records in other arthropods, for example: *Spodoptera littoralis* [49], *Plutella xylostella* [50], *Bactrocera minax* [51], and *Aedes albopictus* [52].

Regarding the differences in bacterial community composition within the gut and gonads, we observe that the relative abundance of certain bacterial taxa shows no significant differences between the female and male gut as well as between the ovaries and testes in each spider species. This contrasts with previous research on wolf spiders (*Pardosa astrigera*), which reported significant differences in gut bacterial composition between females and males [53]. While the three spider species in our study inhabit the same geographical area and share similar food resources, the gut bacterial composition of *E. cavaleriei* differs from that of *L. cornutus* and *P. pseudoannulata*. In the same habitat, the microbial composition of individual spiders may exhibit certain similarities, but this similarity is not absolute. Previous studies have indicated that spiders from the same habitat may harbor different dominant symbiotic microbial communities [54]. We found that individuals of *E. cavaleriei* were infected with *Wolbachia*. *Wolbachia* is primarily transmitted through the maternal lineage [34,55], and its active invasion into host tissues and influence on host biology processes may promote the frequency of infection within host populations [56,57]. In this study, the relevant abundance of *Wolbachia* shows differences in the bacterial communities of female and male samples in *E. cavaleriei*, which markedly caused the differences in bacterial communities between female and male individuals of *E. cavaleriei*. In *L. cornutus* samples, *Spiroplasma* dominated the bacterial communities, and different abundances of *Spiroplasma* in female and male samples resulted in the differences in female and male bacterial communities. In *P. pseudoannulata*, the abundances of endosymbionts *Wolbachia* and *Spiroplasma* in both the gut and reproductive glands are ≤1%, and only a few taxa in the bacterial communities show abundance differences in female and male samples.

While *Wolbachia* and *Spiroplasma* were the dominant endosymbionts in *E. cavaleriei* and *L. cornutus*, respectively, in this study, both gut and gonad samples were pooled from five individual spiders. This suggests that not all individual spiders were infected with *Wolbachia* or *Spiroplasma* endosymbionts. The PCA plot reveals a high similarity in the bacterial communities among the gut and gonads of both male and female *L. cornutus* (Figure 2). However, for *L. cornutus*, one of the three replicates on the PCA plot appears as an outlier, possibly due to variations in the infection status and abundance of *Spiroplasma* endosymbiont bacteria within each individual among the mixed group of five spiders. To address this issue, one could consider increasing the number of individuals in the mixed samples or incorporating more biological replicates in future studies.

The gut structure of spiders differs from that of other arthropods. The foregut of the spider is distributed in the carapace of the spider and diverges from the sucking stomach to the eight legs of the spider to form a blind end at the base of the spider. The foregut passes through the pedicel backwards, and the mid-hind gut is located in the abdomen of the spider. The intestinal wall of the mid-hindgut has a large number of attached branches, and the intestinal texture is fragile. It is noteworthy that the gonads of spiders are positioned below the gut and are closely associated with it [58,59]. Additionally, pathogens often utilize the host’s gut epithelium as a route of entry for systemic infection [3]. This increases the potential for the transmission of gut microbiota to the gonads. Similar situations have been confirmed in other arthropods. In *Galleria mellonella* (Lepidoptera, Pyralidae), it has been revealed that gut bacteria can traverse through the intestinal epithelium and enter the hemocoel, accumulating within the ovaries and eventually being deposited into eggs [60]. The gut bacteria of *Bactrocera dorsalis Hendel* (Diptera, Tephritidae) have also been found to spread to its reproductive system [61], impacting the development of eggs [62]. In this study, we found that within the same spider species, there is almost no significant difference in the abundance of bacterial genera between the female spider’s gut and ovaries. However, some differences in the abundance of bacterial genera were observed between the male spider’s gut and testes. Furthermore, we also observed that, compared to the spider’s reproductive glands, the bacterial communities in the gut exhibited higher diversity and relative abundance. This is in contrast to the findings in *Bactrocera minax* (Diptera, Tephritidae), where the study revealed a higher diversity of gonad bacteria compared to gut bacteria [51]. In the study, it was observed that the gut bacterial communities of the same spider species exhibited a similar trend to the ovarian bacterial communities. This could suggest a potential underlying pathway for the transmission between gut and gonad bacteria in female spiders. It is worth noting that despite the highly unique structure of the spider gut, which contains numerous small branches and Malpighian tubes leading to the body cavity, the question of whether the spider gut can serve as a pathway for gut bacteria to transfer to the gonad, as well as the specific mechanisms of this transfer process, still requires further in-depth research for verification.

The anatomy of the spider gut and gonad forms the foundation for in-depth investigations into endosymbiotic bacteria within spider bodies. The diversity in gut structure and gonad characteristics among different spider species may be attributed to factors such as food sources, body size, developmental stage, and reproductive capacity. For example, the dung sac of *E. cavaleriei* and *L. cornutus* appears brownish-yellow, whereas in *P. pseudoannulata*, it is white. This difference in coloration may be associated with variations in their dietary preferences. The midgut of *P. pseudoannulata* is shorter compared to that of *E. cavaleriei* and *L. cornutus*, which might be related to their body sizes. The eggs of *L. cornutus* and *P. pseudoannulata* are white and have elongated ovaries, while the eggs of *E. cavaleriei* are light yellow and have rounded ovaries. This difference might be related to the developmental stage of each spider and whether the eggs are fertilized. The testes of *L. cornutus* and *P. pseudoannulata* are relatively slender, similar in thickness to their gut, while the testes of *E. cavaleriei* are larger and more robust. The morphology and size of spider testes might be associated with their sperm storage capacity. However, these findings still require further experiments for validation and exploration.

Furthermore, in comparing the microbial diversity of *E. cavaleriei* and *L. cornutus*, both belonging to the Araneidae family and sharing similar predation strategies, we can infer that there may not be a strong correlation between a spider’s predation strategy and the diversity of its gut and gonad microbial communities. Additionally, it can be concluded that being in the same family may not be a necessary condition to determine whether the gut and gonad microbial communities of spiders are similar. Kennedy, S. R. [20] conducted research that unveiled a potential connection between the diversity of microbial communities in spider guts and the types of prey they consume. Therefore, for a more comprehensive understanding of the microbial communities in spiders of different species or with different predation strategies, future research should involve extensive sampling of various spider species in the same habitat for validation.

## Figures and Tables

**Table 1:** Information on samples used in this study.

Spider Species	Tissue	Sample ID
*E. cavaleriei*	Female gut	Egf
	Male gut	Egm
	Ovary	Eo
	Testis	Et
*L. cornutus*	Female gut	Lgf
	Male gut	Lgm
	Ovary	Lo
	Testis	Lt
*P. pseudoannulata*	Female gut	Pgf
	Male gut	Pgm
	Ovary	Po
	Testis	Pt

**Table 2:** Richness and diversity estimation of the gut and gonadal bacteria communities in three spider species (*E. cavaleriei*, *L. cornutus*, and *P. pseudoannulata*).

Sample ID	Number of OTUs	Community Diversity		Species Richness	
		Shannon	Simpson	Ace	Chao1
Egf	958	5.35 ± 0.16	0.01 ± 0.00	806.45 ± 62.59	709.32 ± 98.94
Egm	902	5.34 ± 0.03	0.01 ± 0.00	803.78 ± 48.88	743.17 ± 21.24
Eo	853	0.85 ± 0.03	0.79 ± 0.01	1006.97 ± 111.60	798.71 ± 29.12
Et	1229	3.17 ± 0.52	0.31 ± 0.10	920.92 ± 37.22	910.88 ± 45.84
Lgf	1242	5.44 ± 0.20	0.02 ± 0.00	873.03 ± 64.70	869.33 ± 64.47
Lgm	1208	5.47 ± 0.06	0.01 ± 0.00	846.85 ± 41.92	849.58 ± 37.85
Lo	1311	5.05 ± 0.43	0.05 ± 0.03	888.44 ± 64.50	880.04 ± 71.39
Lt	1293	5.53 ± 0.01	0.01 ± 0.00	868.51 ± 9.29	868.24 ± 11.28
Pgf	1469	5.67 ± 0.07	0.01 ± 0.00	1024.92 ± 64.37	1028.60 ± 65.46
Pgm	1353	5.57 ± 0.10	0.01 ± 0.00	890.14 ± 69.14	894.97 ± 67.27
Po	1469	5.63 ± 0.16	0.01 ± 0.00	982.96 ± 154.58	989.46 ± 154.28
Pt	1323	5.46 ± 0.12	0.01 ± 0.00	1033.31 ± 77.99	1004.71 ± 37.93

Note: Data are means ± SE. The differences are based on the Student’s *t*-test. The specific names corresponding to the sample ID are displayed in Table 1.

**Table 3:** The relative abundance of bacterial taxa in the female and male guts of three different spider species (Top 30).

Phylum/Genus	Abundance (%)					
	Egf	Egm	Lgf	Lgm	Pgf	Pgm
Proteobacteria	88.70 ± 3.37 a	88.20 ± 3.08 a	15.38 ± 2.69 b	14.30 ± 1.37 b	17.20 ± 1.18 b	12.30 ± 0.56 b
*Wolbachia*	86.20 ± 4.45 a	86.10 ± 3.56 a	0.10 ± 0.01 b	0.08 ± 0.01 b	0.12 ± 0.03 b	0.01 ± 0.01 b
*Massilia*	0.45 ± 0.13 a	0.46 ± 0.12 a	0.34 ± 0.09 a	0.56 ± 0.11 a	3.30 ± 0.33 b	3.85 ± 0.22 b
*Desulfovibrio*	0.22 ± 0.05 a	0.25 ± 0.10 a	0.36 ± 0.09 a	0.28 ± 0.06 a	1.16 ± 0.10 b	1.58 ± 0.11 b
*Escherichia-Shigella*	0.12 ± 0.04 a	0.15 ± 0.05 a	0.31 ± 0.12 a	0.29 ± 0.05 a	0.75 ± 0.08 b	0.93 ± 0.04 b
*Halomonas*	0.06 ± 0.02 a	0.10 ± 0.03 a	0.23 ± 0.00 b	0.43 ± 0.01 b	0.75 ± 0.05 c	0.68 ± 0.07 c
Firmicutes	4.18 ± 1.25 a	4.22 ± 0.96 a	22.42 ± 2.53 b	29.90 ± 1.09 b	29.00 ± 2.49 b	28.62 ± 1.29 b
*Virgibacillus*	0.70 ± 0.21 a	0.69 ± 0.15 a	4.35 ± 0.10 b	5.28 ± 0.06 b	4.66 ± 0.38 b	5.22 ± 0.25 b
*Lachnospiraceae_NK4A136_group*	0.54 ± 0.13 a	0.62 ± 0.17 a	2.90 ± 0.63 b	3.98 ± 0.05 c	3.96 ± 0.36 c	4.42 ± 0.37 c
*uncultured_bacterium_f_Lachnospiraceae*	0.18 ± 0.04 a	0.22 ± 0.06 a	1.16 ± 0.29 b	1.33 ± 0.30 b	1.33 ± 0.25 b	1.42 ± 0.19 b
*Lactobacillus*	0.16 ± 0.05 a	0.12 ± 0.05 a	0.67 ± 0.19 ab	1.11 ± 0.10 ab	1.49 ± 0.41 b	1.10 ± 0.10 ab
*Ruminiclostridium_9*	0.13 ± 0.05 a	0.15 ± 0.04 a	0.97 ± 0.12 b	0.98 ± 0.08 b	1.01 ± 0.09 b	1.04 ± 0.04 b
*uncultured_bacterium_f_Ruminococcaceae*	0.13 ± 0.04 a	0.14 ± 0.01 a	0.70 ± 0.10 c	1.25 ± 0.09 b	0.89 ± 0.03 c	1.05 ± 0.12 c
*Bacillus*	0.34 ± 0.20 ab	0.13 ± 0.01 a	0.71 ± 0.12 ab	0.88 ± 0.18 b	0.87 ± 0.16 b	0.87 ± 0.01 b
*Ruminococcaceae_UCG-005*	0.12 ± 0.05 a	0.13 ± 0.03 a	0.76 ± 0.19 b	0.90 ± 0.13 b	0.84 ± 0.07 b	0.94 ± 0.15 b
*Ruminococcaceae_UCG-014*	0.13 ± 0.04 a	0.18 ± 0.06 a	0.74 ± 0.14 b	0.89 ± 0.08 b	0.86 ± 0.11 b	0.79 ± 0.06 b
*Quinella*	0.13 ± 0.03 a	0.14 ± 0.04 a	0.79 ± 0.17 b	0.86 ± 0.07 b	0.74 ± 0.16 b	0.67 ± 0.07 b
*Ruminococcus_1*	0.10 ± 0.03 a	0.13 ± 0.03 a	0.58 ± 0.09 b	0.83 ± 0.09 b	0.63 ± 0.10 b	0.72 ± 0.05 b
*Turicibacter*	0.09 ± 0.03 a	0.09 ± 0.01 a	0.57 ± 0.19 b	0.67 ± 0.01 b	0.69 ± 0.04 b	0.65 ± 0.05 b
Bacteroidetes	3.16 ± 0.91 a	3.44 ± 1.04 a	18.75 ± 3.22 b	22.50 ± 0.99 b	24.40 ± 1.00 b	2.10 ± 0.50 a
*Prevotellaceae_UCG-003*	0.66 ± 0.18 a	0.82 ± 0.24 a	4.86 ± 0.28 b	5.27 ± 0.36 b	5.13 ± 0.56 b	6.35 ± 0.44 b
*Bacteroidales_S24-7_group*	0.65 ± 0.17 a	0.67 ± 0.26 a	3.78 ± 0.14 b	5.02 ± 0.02 c	4.55 ± 0.13 bc	4.49 ± 0.18 bc
*Prevotella_9*	0.49 ± 0.14 a	0.55 ± 0.21 a	2.88 ± 0.19 b	3.19 ± 0.02 b	3.22 ± 0.33 b	3.24 ± 0.21 b
*Bacteroides*	0.30 ± 0.08 a	0.30 ± 0.09 a	1.37 ± 0.08 b	1.86 ± 0.01 b	1.84 ± 0.18 b	1.89 ± 0.33 b
*Prevotellaceae_NK3B31_group*	0.21 ± 0.05 a	0.26 ± 0.04 a	1.31 ± 0.30 b	1.71 ± 0.25 b	1.44 ± 0.09 b	1.65 ± 0.26 b
*Prevotella_1*	0.10 ± 0.03 a	0.10 ± 0.01 a	0.55 ± 0.02 ab	0.95 ± 0.19 b	0.78 ± 0.17 b	0.99 ± 0.06 b
Actinobacteria	1.69 ± 0.49 a	1.80 ± 0.50 a	10.37 ± 1.82 b	12.40 ± 0.64 b	11.40 ± 0.14 b	5.27 ± 0.08 a
*uncultured_bacterium_c_MB-A2-108*	0.22 ± 0.05 a	0.26 ± 0.09 a	1.25 ± 0.06 b	1.83 ± 0.35 b	1.41 ± 0.18 b	1.81 ± 0.19 b
*Rubrobacter*	0.12 ± 0.02 a	0.13 ± 0.02 a	0.62 ± 0.03 b	0.85 ± 0.12 b	0.74 ± 0.09 b	0.86 ± 0.10 b
*Geodermatophilus*	0.08 ± 0.04 a	0.11 ± 0.06 a	0.55 ± 0.16 ab	0.74 ± 0.07 b	0.55 ± 0.09 ab	0.84 ± 0.14 b
*Streptomyces*	0.09 ± 0.02 a	0.11 ± 0.11 a	0.58 ± 0.22 a	0.46 ± 0.07 a	0.56 ± 0.14 a	0.66 ± 0.06 a
Tenericutes	0.10 ± 0.04 a	0.09 ± 0.03 a	4.46 ± 0.65 a	6.04 ± 3.86 a	0.47 ± 0.09 a	1.01 ± 0.77 a
*Spiroplasma*	0.00 ± 0.00 a	0.00 ± 0.00 a	18.73 ± 16.25 a	5.55 ± 5.55 a	0.01 ± 0.01 a	0.00 ± 0.00 a
Spirochaetae	0.78 ± 0.18 a	0.91 ± 0.22 a	4.46 ± 0.28 b	5.87 ± 0.12 c	5.19 ± 0.14 bc	5.27 ± 0.12 bc
*Treponema_2*	0.78 ± 0.18 a	0.91 ± 0.22 a	4.44 ± 0.10 b	5.86 ± 0.01 c	5.01 ± 0.55 bc	5.59 ± 0.04 bc
Cyanobacteria	0.43 ± 0.13 a	0.52 ± 0.12 a	2.83 ± 0.49 b	3.71 ± 0.19 b	3.31 ± 0.10 b	2.70 ± 0.87 b
*Microcoleus*	0.28 ± 0.09 a	0.32 ± 0.07 a	1.74 ± 0.16 b	2.36 ± 0.02 bc	2.16 ± 0.15 bc	2.71 ± 0.16 c

Note: Data are shown as Mean ± SE; The data were compared using the Bonferroni test, which tests for differences between different gut groups in phylum and genus level; Values with different letters indicate a significant difference (*p* < 0.05). The specific names corresponding to the sample ID are displayed in Table 1.

**Table 4:** The relative abundance of bacterial taxa in the ovary and testis of three different spider species (Top 30).

Phylum/Genus	Abundance (%)					
	Eo	Et	Lo	Lt	Po	Pt
Proteobacteria	93.08 ± 0.33 a	65.19 ± 7.88 b	13.60 ± 2.15 c	14.40 ± 0.48 c	14.60 ± 1.07 c	8.82 ± 4.36 c
*Wolbachia*	91.94 ± 0.27 a	57.31 ± 9.30 b	0.01 ± 0.00 c	0.00 ± 0.00 c	0.46 ± 0.43 c	0.79 ± 0.34 c
*Massilia*	0.29 ± 0.03 a	1.45 ± 0.36 ab	2.97 ± 0.09 ab	3.18 ± 0.71 b	2.66 ± 0.78 ab	1.23 ± 0.79 ab
*Desulfovibrio*	0.14 ± 0.03 a	0.73 ± 0.25 ab	1.39 ± 0.11 ab	1.68 ± 0.41 b	1.14 ± 0.22 ab	0.76 ± 0.32 ab
*Escherichia-Shigella*	0.07 ± 0.02 a	0.43 ± 0.03 ab	0.80 ± 0.05 ab	1.20 ± 0.13 b	0.73 ± 0.18 ab	0.52 ± 0.40 ab
Firmicutes	2.47 ± 0.13 a	13.50 ± 2.83 b	24.70 ± 2.50 c	32.60 ± 0.50 c	30.60 ± 2.59 c	26.60 ± 1.43 c
*Virgibacillus*	0.47 ± 0.02 a	2.56 ± 0.67 ab	4.36 ± 0.24 ab	4.99 ± 0.38 b	4.57 ± 1.13 ab	2.04 ± 1.51 ab
*Lachnospiraceae_NK4A136_group*	0.35 ± 0.01 a	1.82 ± 0.33 ab	3.69 ± 0.41 b	4.73 ± 0.50 b	3.78 ± 0.87 b	1.75 ± 0.93 ab
*uncultured_bacterium_f_Lachnospiraceae*	0.08 ± 0.01 a	0.71 ± 0.19 ab	1.17 ± 0.09 bc	1.80 ± 0.15 c	1.34 ± 0.29 bc	0.73 ± 0.23 ab
*uncultured_bacterium_f_Ruminococcaceae*	0.06 ± 0.00 a	0.51 ± 0.14 ab	0.81 ± 0.09 ab	1.15 ± 0.27 b	0.96 ± 0.19 ab	0.70 ± 0.27 ab
*Ruminococcaceae_NK4A214_group*	0.04 ± 0.01 a	0.29 ± 0.08 a	0.49 ± 0.09 ab	0.69 ± 0.17 ab	1.05 ± 0.30 ab	1.62 ± 0.45 b
*Succiniclasticum*	0.02 ± 0.01 a	0.04 ± 0.03 a	0.12 ± 0.11 a	0.06 ± 0.03 a	0.96 ± 0.86 a	3.03 ± 1.50 a
*Bacillus*	0.09 ± 0.02 a	0.50 ± 0.10 ab	0.81 ± 0.10 ab	1.31 ± 0.12 b	1.07 ± 0.31 b	0.39 ± 0.21 b
*Ruminococcaceae_UCG-014*	0.04 ± 0.00 a	0.47 ± 0.07 ab	0.88 ± 0.21 ab	1.16 ± 0.24 b	0.69 ± 0.01 b	0.70 ± 0.08 ab
*Ruminiclostridium_9*	0.11 ± 0.01 a	0.48 ± 0.19 ab	0.86 ± 0.06 ab	1.24 ± 0.11 b	0.93 ± 0.29 ab	0.34 ± 0.25 ab
*Lactobacillus*	0.10 ± 0.06 a	0.35 ± 0.05 ab	0.69 ± 0.12 ab	1.04 ± 0.23 b	0.75 ± 0.19 ab	0.41 ± 0.27 ab
*Ruminococcaceae_UCG-005*	0.07 ± 0.01 a	0.36 ± 0.12 a	0.64 ± 0.11 ab	1.13 ± 0.11 b	0.71 ± 0.12 ab	0.36 ± 0.23 a
Bacteroidetes	2.04 ± 0.11 a	11.10 ± 2.62 a	20.40 ± 2.77 ab	24.60 ± 0.95 ab	29.80 ± 5.28 ab	47.70 ± 12.30 b
*Prevotellaceae_UCG-003*	0.49 ± 0.01 a	2.46 ± 0.52 ab	5.25 ± 0.54 b	6.11 ± 1.02 b	5.07 ± 1.12 b	3.13 ± 0.73 ab
*Bacteroidales_S24-7_group*	0.42 ± 0.05 a	2.60 ± 0.62 ab	3.92 ± 0.54 ab	5.66 ± 0.93 b	4.30 ± 1.23 ab	2.06 ± 1.33 ab
*Prevotella_1*	0.10 ± 0.03 a	0.43 ± 0.10 a	1.05 ± 0.32 a	0.81 ± 0.22 a	3.69 ± 2.92 a	11.3 ± 5.35 a
*Rikenellaceae_RC9_gut_group*	0.06 ± 0.03 a	0.24 ± 0.09 a	0.29 ± 0.14 a	0.37 ± 0.13 a	2.76 ± 2.45 a	10.10 ± 4.91 a
*uncultured_rumen_bacterium*	0.05 ± 0.01 a	0.16 ± 0.08 a	0.23 ± 0.11 a	0.26 ± 0.03 a	2.96 ± 2.67 a	9.62 ± 4.69 a
*Prevotella_9*	0.27 ± 0.03 a	1.58 ± 0.49 ab	2.85 ± 0.32 ab	3.63 ± 0.16 b	3.06 ± 0.79 ab	1.22 ± 0.90 ab
*Bacteroides*	0.00 ± 0.00 a	0.19 ± 0.07 a	1.66 ± 0.30 a	2.48 ± 0.59 a	1.58 ± 0.38 a	1.48 ± 1.21 a
*Prevotellaceae_NK3B31_group*	0.16 ± 0.01 a	0.82 ± 0.22 ab	1.48 ± 0.29 b	1.54 ± 0.27 b	1.76 ± 0.12 b	1.38 ± 0.05 b
*Bacteroidales_BS11_gut_group*	0.02 ± 0.01 a	0.04 ± 0.01 a	0.05 ± 0.03 a	0.07 ± 0.04 a	0.86 ± 0.76 a	3.02 ± 1.46 a
Actinobacteria	1.11 ± 0.08 a	5.53 ± 1.13 ab	11.00 ± 1.01 b	12.60 ± 0.98 b	11.00 ± 2.09 b	4.93 ± 3.20 ab
*uncultured_bacterium_c_MB-A2-108*	0.15 ± 0.03 a	0.71 ± 0.22 a	1.61 ± 0.20 a	1.56 ± 0.15 a	1.51 ± 0.40 a	0.67 ± 0.47 a
Spirochaetae	0.43 ± 0.03 a	2.60 ± 0.45 ab	4.93 ± 0.43 b	5.42 ± 0.48 b	4.19 ± 0.96 b	2.55 ± 1.00 ab
*Treponema_2*	0.43 ± 0.03 a	2.60 ± 0.45 ab	4.93 ± 0.43 b	5.42 ± 0.84 b	4.16 ± 0.99 ab	2.47 ± 1.04 ab
Tenericutes	0.05 ± 0.01 a	0.26 ± 0.06 a	15.90 ± 8.86 a	1.85 ± 0.59 a	0.55 ± 0.09 a	0.39 ± 0.02 a
*Spiroplasma*	0.00 ± 0.00 a	0.00 ± 0.00 a	15.30 ± 9.00 a	1.07 ± 0.91 a	0.00 ± 0.00 a	0.00 ± 0.00 a
Cyanobacteria	0.31 ± 0.01 a	1.52 ± 0.19 a	3.20 ± 0.3 bc	3.50 ± 0.08 b	2.76 ± 0.63 bc	1.35 ± 0.65 a
*Microcoleus*	0.21 ± 0.02 a	1.07 ± 0.14 ab	2.11 ± 0.21 b	2.22 ± 0.16 b	1.80 ± 0.46 b	0.78 ± 0.44 ab
Gemmatimonadetes	0.12 ± 0.01 a	0.73 ± 0.13 ab	1.83 ± 0.28 b	1.90 ± 0.07 b	1.62 ± 0.36 b	0.71 ± 0.47 ab
*uncultured_bacterium_f_Longimicrobiaceae*	0.05 ± 0.01 a	0.35 ± 0.07 ab	0.81 ± 0.08 ab	1.03 ± 0.27 b	0.73 ± 0.16 ab	0.33 ± 0.26 ab

Data are shown as Mean ± SE; The data were compared using the Bonferroni test, which tests for differences between different gonad groups in phylum and genus level; Values with different letters indicate a significant difference (*p* < 0.05). The specific names corresponding to the sample ID are displayed in Table 1.

**Table 5:** Analysis of the difference in bacterial relative abundance in the gut and gonad of three different spider species.

Phylum/Genus	Comparison of Differences in Relative Abundance (*p*-Value)					
	Egf/Eo	Egm/Et	Lgf/Lo	Lgm/Lt	Pgf/Po	Pgm/Pt
Proteobacteria	-	*	-	-	-	-
*Wolbachia*	-	*	-	-	-	-
*Massilia*	-	*	-	-	-	*
*Desulfovibrio*	-	-	-	-	-	-
*Escherichia-Shigella*	-	**	-	*	-	-
Firmicutes	-	*	-	-	-	-
*Virgibacillus*	-	*	-	-	-	-
*Lachnospiraceae_NK4A136_group*	-	*	-	*	-	*
*uncultured_bacterium_f_Lachnospiraceae*	*	-	-	-	-	-
*Lactobacillus*	-	*	-	-	-	-
*Ruminiclostridium_9*	-	-	-	-	-	*
*uncultured_bacterium_f_Ruminococcaceae*	-	*	-	-	-	-
*Bacillus*	-	*	-	-	-	*
*Ruminococcaceae_UCG-005*	-	-	-	-	-	-
*Ruminococcaceae_UCG-014*	*	*	-	-	-	-
Bacteroidetes	-	-	-	-	-	-
*Prevotellaceae_UCG-003*	-	*	-	-	-	*
*Bacteroidales_S24-7_group*	-	*	-	-	-	-
*Prevotella_9*	-	-	-	*	-	-
*Bacteroides*	-	**	-	-	-	-
*Prevotellaceae_NK3B31_group*	-	*	-	-	-	-
*Prevotella_1*	-	*	-	-	-	-
Actinobacteria	-	*	-	-	-	*
*uncultured_bacterium_c_MB-A2-108*	-	-	-	-	-	-
Tenericutes	-	-	-	-	-	-
*Spiroplasma*	-	-	-	-	-	-
Spirochaetae	-	*	-	-	-	*
*Treponema_2*	-	*	-	-	-	*
Cyanobacteria	-	**	-	-	-	*
*Microcoleus*	-	**	-	-	-	*

Note: The table displays the shared bacterial genera among the top 30 in terms of relative abundance in the gut and gonad. “*” indicates a significant difference, “**” indicates a highly significant difference, and “-” indicates no difference. The specific names corresponding to the sample ID are displayed in Table 1.

## Data Availability

All data used in this paper are available within the text.
